# Hypernatremia Caused by Saltwater Emesis in a Case of Neem Powder Poisoning

**DOI:** 10.7759/cureus.89685

**Published:** 2025-08-09

**Authors:** Krishnakar Reddy Alla, Rusheeka Pulluri

**Affiliations:** 1 Internal Medicine, Sri Venkata Sai (SVS) Medical College, Mahbubnagar, IND

**Keywords:** community-acquired hypernatremia, real-world education, saltwater, self-poisoning, suicide prevention

## Abstract

Table salt is widely available and, in some cases, inappropriately used as an emetic in poisoning incidents, both in clinical and domestic settings. However, saltwater-induced emesis can lead to severe hypernatremia, which, although rare, carries a high risk of morbidity and mortality. Early recognition, prompt diagnosis, and urgent management are essential to prevent irreversible neurological injury or death. We report a case of severe hypernatremia (serum sodium 187 mmol/L) following saltwater-induced emesis in a young woman with suspected neem powder poisoning, in whom timely intervention led to complete recovery. Clinical features, management strategies, and the urgent need to eliminate this dangerous practice are discussed.

## Introduction

Sodium chloride is an essential dietary electrolyte; however, acute ingestion of large quantities can cause life-threatening hypernatremia, defined as a serum sodium concentration exceeding 145 mmol/L [1,2]. Historically, concentrated saltwater was used as an emetic, an agent given to provoke vomiting, but this practice is now strongly discouraged due to well-documented salt toxicity [3].

Most modern cases of hypernatremia occur in elderly or hospitalized patients, often as a result of dehydration or excessive intravenous saline administration [4,5]. In contrast, salt-induced hypernatremia from oral intake is rare and under-reported in otherwise healthy adults [6,7]. When it does occur, published series suggest a predilection for young women with psychiatric illness or those engaging in ritualistic or alternative-medicine practices [8]. Unfortunately, concentrated saltwater is still sometimes used as misguided first aid for poisoning in resource-limited or rural settings [3].

Neem (*Azadirachta indica*) is an evergreen tree native to the Indian subcontinent. Its leaves and seeds are ground into neem powder, a traditional remedy promoted for antiparasitic, hypoglycemic, and contraceptive effects. Although generally well tolerated in small amounts, high-dose ingestion has been linked to gastrointestinal irritation, metabolic acidosis, and seizures [9].

We report a case of profound hypernatremia (serum sodium 187 mmol/L) precipitated by the administration of concentrated saltwater to induce emesis after neem powder ingestion in a young woman. This case underscores the catastrophic potential of saltwater emesis and the urgent need for public and professional education to eliminate this dangerous practice.

## Case presentation

A 24-year-old woman ingested approximately two teaspoons of neem (*Azadirachta indica*) powder in a suicide attempt. About 30 minutes later, clinic staff administered an undetermined volume of concentrated saltwater (~200 mL of saturated solution) to induce emesis. During ambulance transfer, approximately 90 minutes after ingestion, she became unresponsive.

On arrival at the emergency department (~2 hours post-ingestion), her Glasgow Coma Scale score was E1 V1 M1, blood pressure 100/70 mm Hg, pulse 118 beats per minute, and SpO₂ 45% on room air. She was intubated within 10 minutes and underwent gastric lavage until the effluent was clear.

Initial laboratory testing and electrolyte profile are shown in Table 1. Treatment commenced 20 minutes after arrival with intravenous 5% dextrose at 1 mL/kg/hour and free water via Ryle’s tube at 100 mL/hour. A non-contrast head CT performed after stabilization showed no intracranial hemorrhage, cerebral edema, or mass effect.

**Table 1:** Laboratory results on admission Initial laboratory investigations revealed profound hypernatremia and other electrolyte abnormalities.

Parameter	Result	Reference range	Unit
Serum sodium	187	135–145	mmol/L
Serum potassium	3.4	3.5–5.1	mmol/L
Serum chloride	145	98–107	mmol/L
Serum bicarbonate	16	22–28	mmol/L
Blood urea nitrogen	9	2.5–7.1	mmol/L
Serum creatinine	72	44–80	µmol/L
Serum glucose	6.2	3.9–6.1	mmol/L
Arterial pH	7.26	7.35–7.45	–
Partial pressure of carbon dioxide	34	35–45	mmHg
Partial pressure of oxygen	48	80–100	mmHg
Oxygen saturation	45	>95	%

**Figure 1:**
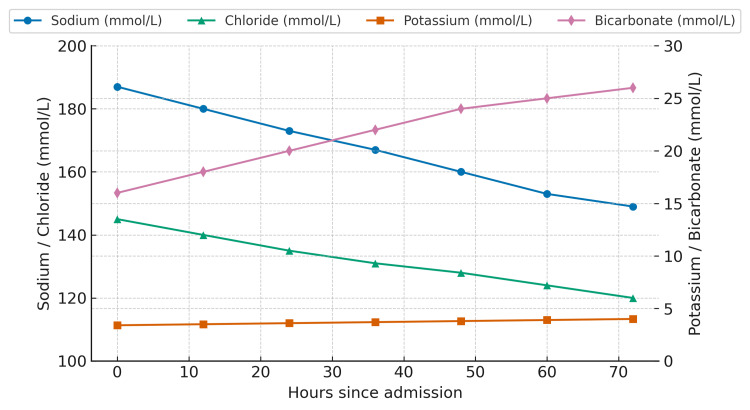
Serum sodium trend during hospitalization Serial serum sodium levels of the patient who had acute saltwater poisoning, demonstrating gradual correction: 187 mmol/L (admission), 180 (12 hours), 173 (24 hours), 167 (36 hours), 161 (48 hours), 155 (60 hours), and 149 mmol/L (72 hours).

Serial monitoring showed a gradual reduction in serum sodium to 149 mmol/L over 72 hours (Figure 1), accompanied by parallel improvement in mental status. She was extubated and discharged without neurological deficits.

## Discussion

Hypernatremia from acute salt poisoning is extremely rare, largely because the unpleasant taste of salt makes large-volume ingestion difficult [7,10,11]. However, saltwater emesis, still practiced in some traditional and rural settings, can cause severe or fatal hypernatremia [3,12]. Campbell and Train [1] estimated the adult lethal dose as less than four tablespoons, or about 72 g of sodium chloride.

Massive salt ingestion rapidly elevates serum sodium and plasma osmolality, driving water out of neurons. This causes cerebral dehydration, neuronal shrinkage, and increases the risk of intracranial hemorrhage, seizures, coma, and sometimes osmotic demyelination [7,12-14]. Paradoxically, cerebral edema may occur after overly rapid correction, especially if the hypernatremia is chronic [15,16].

Acute symptomatic hypernatremia (<48 hours) from salt poisoning should be corrected promptly with hypotonic fluid, either free water orally or via nasogastric tube, or intravenous 5% dextrose [17,18]. The target is a sodium reduction of 0.5-1 mmol/L per hour, with close monitoring for complications [17]. Chronic hypernatremia, in contrast, requires slower correction (<0.5 mmol/L/h) to avoid cerebral edema [4,17]. Hemodialysis may be indicated in cases with renal failure or volume overload [4,17]. In this case, a non-contrast head CT performed immediately after hemodynamic stabilization showed no cerebral edema or hemorrhage, consistent with truly acute (<48 h) hypernatremia and the absence of raised intracranial pressure [15,16].

Because the presentation was acute and symptomatic, we aimed for rapid correction with free water replacement. The free water deficit, calculated by the Adrogué-Madias formula (0.6 × body mass × [(Na/140) - 1]), was approximately 5 L. We initiated 5% dextrose at 1 mL/kg/h and enteral water at 100 mL/h. Serum sodium declined at 0.8-0.9 mmol/L/h, within the recommended target for acute hypernatremia [17,18]. Dialysis was not required, as urine output remained >1 mL/kg/h and the patient stayed euvolemic, meeting criteria for conservative management [4,17].

Saltwater remains promoted as an emetic in some low-resource communities. Recent case reports, including ours, show that this practice can be fatal. Community education and first-responder training are essential to replace this hazardous method with safer poisoning management protocols [19]. This case supports aggressive but controlled sodium correction in acute salt poisoning and strengthens the call to eliminate saltwater emesis as a first-aid measure.

## Conclusions

Salt poisoning is rare but potentially fatal. Saltwater emesis should be strongly discouraged as a home or clinical remedy for poisoning. Prompt recognition and timely intervention can lead to full recovery, as in our patient. Continued public and healthcare education is essential to prevent future cases.
